# Attributable fractions of risk factors for cognitive impairment in the ELSA‐Brasil cohort

**DOI:** 10.1002/alz.092360

**Published:** 2025-01-09

**Authors:** Raphael Machado Castilhos, Vanessa Bielenfeldt Leotti, Natan Feter, Bruce Duncan, Maria Inês Schmidt

**Affiliations:** ^1^ Hospital de Clínicas de Porto Alegre, Porto Alegre, Rio Grande do Sul Brazil; ^2^ Hospital de Clínicas de Porto Alegre, Porto Alegre, Rio Grande de Sul Brazil; ^3^ Universidade Federal do Rio Grande do Sul, Porto Alegre Brazil; ^4^ Postgraduate Program in Epidemiology, Universidade Federal do Rio Grande do Sul, Porto Alegre Brazil; ^5^ Medical School, Universidade Federal do Rio Grande do Sul, Porto Alegre Brazil

## Abstract

**Background:**

Attributable fractions (AFs) of the modifiable risk factors of dementia are usually calculated using population‐based samples. Nevertheless, assessing AF within cohorts offers a more nuanced perspective on the impact of these factors. We aimed to estimate the relative risks (RRs) for cognitive impairment (CI) for eight risk factors for dementia and their respective AF in the Longitudinal Study of Adult Health (ELSA‐Brasil).

**Methods:**

A global cognitive function score was established by averaging Z scores from six standardized tests. Risk factors and sociodemographic variables were collected between 2008 and 2010, while CI (defined as < ‐1.5 SD on the global cognition Z score) was assessed approximately 10 years later. Participants with CI in the baseline were excluded. We calculated the RR for CI of eight risk factors (physical inactivity, depression, tobacco use, excessive alcohol consumption, obesity, low education, diabetes and hypertension) using a robust Poisson model. AFs were computed using the average attributable fraction method.

**Results:**

Of the 15,105 individuals of the ELSA‐Brasil, 10,058 were included in this analysis. At baseline, the median (IQR) age was 50 (44‐56) years and 56.7% (5,704) were women. The incidence of CI was 5.5% (n = 549). RRs for CI were significant for low education (4.37, 3.61‐5.29), hypertension (1.43, 1.21‐1.7), smoking (1.37, 1.09‐1.71), diabetes (1.27, 1.05‐1.54), in addition to male sex (1.31, 1.11‐1.55) and older age (1.07, 1.06‐1.08). Adjusted risks for depression, physical inactivity, excessive alcohol consumption and obesity were not significant. Low education had the highest AF (CI 95%), 14.3% (10.8‐17.7), followed by hypertension, 13.1% (6.8‐19.4), diabetes, 4.3% (0.4‐8.1) and smoking 3.3% (0.5‐6.1). The total AF for CI was 35% (28‐42).

**Conclusions:**

This is the first assessment of AF of the modifiable dementia risk factors in a Brazilian cohort, with RR calculated directly from the sample. In the ELSA‐Brasil, assuming causality, around one‐third of cognitive impairment cases could be prevented if these four risk factors were eliminated.

